# The Role of Multimodality Imaging (CT & MR) as a Guide to the Management of Chronic Coronary Syndromes

**DOI:** 10.3390/jcm13123450

**Published:** 2024-06-13

**Authors:** Luigi Tassetti, Enrico Sfriso, Francesco Torlone, Andrea Baggiano, Saima Mushtaq, Francesco Cannata, Alberico Del Torto, Fabio Fazzari, Laura Fusini, Daniele Junod, Riccardo Maragna, Alessandra Volpe, Nazario Carrabba, Edoardo Conte, Marco Guglielmo, Lucia La Mura, Valeria Pergola, Roberto Pedrinelli, Ciro Indolfi, Gianfranco Sinagra, Pasquale Perrone Filardi, Andrea Igoren Guaricci, Gianluca Pontone

**Affiliations:** 1 Perioperative Cardiology and Cardiovascular Imaging Department, Centro Cardiologico Monzino IRCCS, 20138 Milan, Italy; luigi.tassetti@cardiologicomonzino.it (L.T.); andrea.baggiano@cardiologicomonzino.it (A.B.); saima.mushtaq@cardiologicomonzino.it (S.M.); francesco.cannata@cardiologicomonzino.it (F.C.); fabio.fazzari@cardiologicomonzino.it (F.F.); laura.fusini@cardiologicomonzino.it (L.F.); daniele.junod@cardiologicomonzino.it (D.J.); riccardo.maragna@cardiologicomonzino.it (R.M.); alessandra.volpe@cardiologicomonzino.it (A.V.); 2 Radiology Unit, Department of Medical, Surgical and Health Sciences, University of Trieste, 34127 Trieste, Italy; enrico.sfriso95@gmail.com; 3 Cardiovascular Department, IRCCS Multimedica, 20099 Milan, Italy; francesco.torlone@multimedica.it; 4 Department of Cardiothoracovascular Medicine, Azienda Ospedaliero-Universitaria Careggi, 50134 Florence, Italy; n.carrabba@virgilio.it; 5 Department of Clinical Cardiology and Cardiovascular Imaging, Galeazzi-Sant’Ambrogio Hospital IRCCS, 20157 Milan, Italy; edoardo.conte86@gmail.com; 6 Department of Cardiology, Division of Heart and Lungs, Medical Center Utrecht, Utrecht University, 3584 Utrecht, The Netherlands; m.guglielmo@umcutrecht.nl; 7 Department of Advanced Biomedical Sciences, University Federico II of Naples, 80131 Naples, Italy; lucia.lamura@hotmail.it (L.L.M.); pasquale.perrone@unina.it (P.P.F.); 8 Department of Cardiac, Thoracic and Vascular Sciences and Public Health, University of Padova, 35128 Padova, Italy; valeria.pergola@unipd.it; 9 Cardiac, Thoracic and Vascular Department, University of Pisa, 56124 Pisa, Italy; roberto.pedrinelli@unipi.it; 10 Istituto di Cardiologia, Dipartimento di Scienze Mediche e Chirurgiche, Università degli Studi “Magna Graecia”, 88100 Catanzaro, Italy; indolfi@unicz.it; 11 Cardiology Specialty School, University of Trieste, 34127 Trieste, Italy; gianfranco.sinagra@asugi.sanita.fvg.it; 12 Center for Diagnosis and Treatment of Cardiomyopathies, Cardiovascular Department, Azienda Sanitaria Universitaria Giuliano-Isontina (ASUGI), 34149 Trieste, Italy; 13 Cardiology Unit, Interdisciplinary Department of Medicine, University of Bari Aldo Moro, 70126 Bari, Italy; andrea.guaricci@gmail.com; 14 Department of Biomedical, Surgical and Dental Sciences, University of Milan, 20122 Milan, Italy

**Keywords:** chronic coronary syndrome, stress cardiac magnetic resonance, coronary computed tomography angiography

## Abstract

Chronic coronary syndrome (CCS) is one of the leading cardiovascular causes of morbidity, mortality, and use of medical resources. After the introduction by international guidelines of the same level of recommendation to non-invasive imaging techniques in CCS evaluation, a large debate arose about the dilemma of choosing anatomical (with coronary computed tomography angiography (CCTA)) or functional imaging (with stress echocardiography (SE), cardiovascular magnetic resonance (CMR), or nuclear imaging techniques) as a first diagnostic evaluation. The determinant role of the atherosclerotic burden in defining cardiovascular risk and prognosis more than myocardial inducible ischemia has progressively increased the use of a first anatomical evaluation with CCTA in a wide range of pre-test probability in CCS patients. Functional testing holds importance, both because the role of revascularization in symptomatic patients with proven ischemia is well defined and because functional imaging, particularly with stress cardiac magnetic resonance (s-CMR), gives further prognostic information regarding LV function, detection of myocardial viability, and tissue characterization. Emerging techniques such as stress computed tomography perfusion (s-CTP) and fractional flow reserve derived from CT (FFRCT), combining anatomical and functional evaluation, appear capable of addressing the need for a single non-invasive examination, especially in patients with high risk or previous revascularization. Furthermore, CCTA in peri-procedural planning is promising to acquire greater importance in the non-invasive planning and guiding of complex coronary revascularization procedures, both by defining the correct strategy of interventional procedure and by improving patient selection. This review explores the different roles of non-invasive imaging techniques in managing CCS patients, also providing insights into preoperative planning for percutaneous or surgical myocardial revascularization.

## 1. Introduction

Chronic coronary syndrome (CCS) is one of the leading causes of morbidity worldwide and is associated with a significant economic and societal burden [1,2]. According to the most recent guidelines, CCS includes a heterogeneous group of clinical scenarios, ranging from patients with stable angina or detection of coronary artery disease (CAD) after screening evaluation to patients already revascularized after acute events or elective procedures [2]. In the aforementioned wide range of scenarios, there is still controversy about the clinical settings in which myocardial revascularization is indicated in patients with CCS. Except for the identification of an obstructive lesion involving the so-called “prognostic” vessels (i.e., left main disease or proximal left anterior descending with stenosis >50%), whose revascularization has been demonstrated to carry a clear prognostic benefit [3], the role of coronary interventional procedures in the presence of documented ischemia in reducing the risk of ischemic cardiovascular events or death is doubtful in light of recent evidence [4].

Still more debated is the role of revascularization after the detection of myocardial viability and/or ischemia in the setting of a chronic total occlusion [5,6].

The controversial value of myocardial inducible ischemia in CCS, and the determinant role played by the atherosclerotic burden in defining prognosis, has always given more importance to anatomical evaluation with cardiac computer tomography angiography (CCTA), leading to a progressive extension of the indication of CCTA in a wide range of pre-test probability in patients with CCS [2,7].

Furthermore, CCTA is acquiring more importance in non-invasive planning and guiding complex coronary revascularization procedures [8]. However, functional testing remains fundamentally important, not only because revascularization’s role in symptomatic patients is well-defined for symptom relief when ischemia is demonstrated, but also because functional imaging, particularly stress cardiac magnetic resonance (s-CMR) with tissue characterization, provides additional information beyond ischemia evaluation that is equally important for patient management.

This review encompasses a description of the role of the different non-invasive imaging techniques in the management of patients with CCS, including the choice and the procedural planning of percutaneous or surgical myocardial revascularization.

## 2. The Role of Multimodality Imaging in Identifying Patients Worthy of Revascularization

The evaluation of CAD can be performed using different methods, classified as non-invasive and invasive, i.e., invasive coronary angiography (ICA), or as anatomical, functional, and “mixed”, i.e., combining anatomical and functional assessment, such as CCTA + fractional flow reserve derived from CCTA datasets (FFR-_CT_) and CCTA + cc (s-CTP). The diagnostic approach should be based primarily on the likelihood of disease, as well as availability, center expertise, and patient characteristics [9]. Following the introduction by the 2019 ESC Guidelines on chronic coronary syndromes of the same level of recommendation to non-invasive imaging techniques (CCTA, stress echocardiography (SE), s-CMR, single-photon emission computed tomography (SPECT) and positron emission tomography (PET)) in terms of CAD detection, a wide debate about the dilemma of choosing a first anatomical or first functional evaluation strategy was generated [2]. Actually, despite the wide range of available imaging techniques to detect CAD, there is still a scarce adoption of the guidelines recommendation, especially because of the persistent, widespread use of stress electrocardiography (sECG), instead of non-invasive imaging, followed by invasive coronary angiography (ICA) as the first imaging test [10].

### 2.1. Anatomical Evaluation: Cardiac Computer Tomography Angiography

CCTA is the only non-invasive anatomical evaluation currently validated to detect coronary artery disease [11].

Over the last years, the use of CCTA has robustly increased in both Europe and the USA and is becoming the most common non-invasive imaging modality after SPECT in the UK, with an annual growth rate of 18.5% (period 2012–2019) [12,13]. The “traditional” first recommendation of CCTA in cardiology guidelines was to rule out CAD in patients with low-to-intermediate clinical likelihood, given the high negative predictive value of this method, useful to exclude significant atherosclerosis in patients with a low cardiovascular profile and typical or atypical symptoms [14]. Following this, technological advancements have progressively reduced technical challenges, such as the reduced temporal resolution of 64-section computer tomography (CT) detectors, thanks to dual-tube systems or whole heart coverage detectors, and the beam artifacts for calcifications, thanks to spectral CT techniques.

Those progressions permitted a precise quantification and characterization of the different types of atherosclerotic plaques (both calcified and non-calcified components of coronary atherosclerosis), thanks to dedicated semi-automated and automated tools [15], overcoming CT calcium scoring, which previously provided a poor surrogate of coronary plaque burden [16]. 

Furthermore, CCTA is able to assess specific features of plaques (such as low attenuation plaque, spotty calcification, positive remodeling, and “napkin ring sign”), identifying the so-called “adverse plaque” phenotype, with a demonstrated prognostic role in CCS [17,18,19].

The evidence just cited has also demonstrated the reliability of plaque virtual histology provided by CCTA in determining plaque instability based on the identification of necrotic and lipid components rather than calcific ones. Additionally, the abovementioned high-risk plaque (HRP) features have been shown to be associated with a higher risk of developing acute coronary syndrome, allowing for CCTA-based predictive tools for acute events.

**Figure 1:**
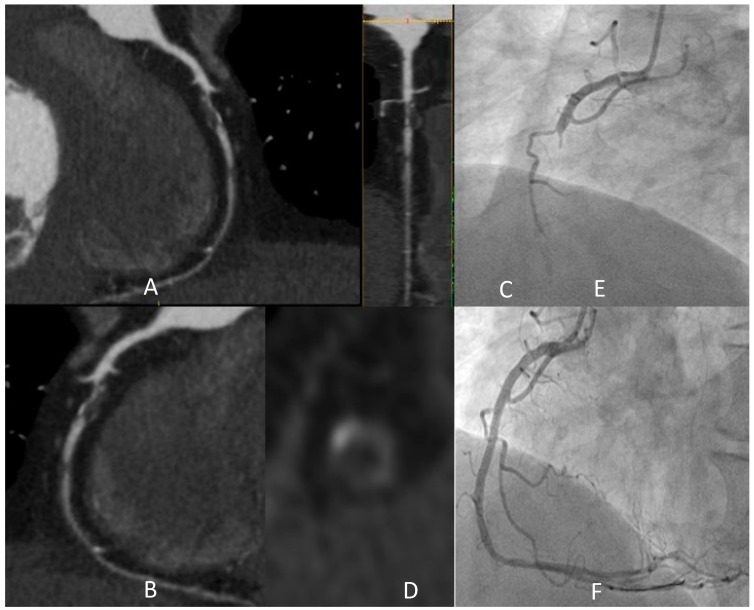
A 73-year-old male active smoker developed typical effort chest pain and CCTA was prescribed. Panel (**A**–**D**) shows a severe stenosis of medium RCA (after the origin of an acute marginal branch) characterized by prevalent lipidic plaque with all the typical HRP features: low attenuation (<30 HU in the central area), “napkin ring sign” (i.e., a low attenuating eccentric part surrounded by a thin, ring-like hyperattenuating rim), focal spotty calcification inside the lesion, and also positive remodeling. One other lipidic subocclusive stenosis is detected in the medium segment. In light of these findings, the patient was recovered to perform ICA in the following days. After an episode of chest pain at rest, urgent ICA was performed (**E**), showing a complete occlusion of the vessel in the segment involved by the abovementioned lesion, treated with PCI with multiple DES implantation (**F**). HRP: “high-risk plaque”; CCTA: coronary computed tomography angiography; DES: drug-eluting stent; HU: Hounsfield units; ICA: invasive coronary angiography; PCI: percutaneous coronary intervention.

Figure 1 shows the detection of a coronary plaque with the typical HRP features, with a clinical scenario witnessing the instability potentially associated with these anatomical findings.

The strong importance of plaque characterization in defining the prognosis of CCS patients with CCTA was first demonstrated by the SCOT-HEART Study [20], a prospective parallel-group, multicenter trial that enrolled 4.146 patients referred for assessment of suspected stable angina, randomly assigned to either standard care plus coronary calcium score and CCTA or standard care alone.

The primary endpoint of the study was the certainty of the diagnosis of angina due to CAD, while the long-term endpoint was a composite of death, myocardial infarction (MI), coronary revascularization procedures, admittance to hospital for chest pain episodes, and cerebrovascular and peripheral vascular disease at a 5-year follow-up (FU).

CCTA significantly reclassified the diagnosis of CAD and of angina due to CAD at short-term FU, influencing changes in planned investigations and treatments: the prescription of antianginal treatments and stress imaging tests were significantly reduced in the CCTA group, while preventive therapy was increased.

CCTA was associated with a non-significant increase in coronary revascularization procedures compared to standard care (11.2% vs. 9.7% (*p* = 0.061), respectively) in the short-term, while no significant differences between the groups were observed in a 5-year FU. 

In the CCTA group, there was a significant reduction of death from coronary heart disease (CHD) or non-fatal MI at a 5-year FU.

Notably, approximately half of the subsequent MI in the CCTA group happened in patients with non-obstructive CAD, and a post-hoc analysis of the SCOT-HEART long FU study showed that beyond the first year, rates of coronary revascularization were higher in those who had received standard care alone, many of which were triggered by MI [21].

These results underlined the importance of CCTA in plaque evaluation, not only in terms of obstructive disease, but also in atherosclerotic burden and plaque phenotypes, suggesting the importance of a target preventive therapy as a main prognostic modifier [22]. Analogous conclusions were acquired from the randomized PROMISE Trial: in 10,003 symptomatic patients with suspected stable disease, initial anatomical testing did not improve clinical outcome compared to functional testing, but anatomical evaluation allowed the identification of a strong association between the adverse plaque phenotype and major adverse cardiovascular events (MACE) [23,24]. 

The clinical implications of plaque characterization were recently confirmed by the PREVENT trial, a recently published multicenter international study. A total of 1606 patients with non-flow-limiting vulnerable coronary plaques were randomly assigned to either percutaneous coronary intervention (PCI) or optimal medical therapy; a 2-year follow-up demonstrated that in cases of non-flow-limiting vulnerable coronary plaques, preventive PCI reduced major adverse cardiac events arising from high-risk vulnerable plaques, compared with optimal medical therapy alone [25]. Although plaque characterization in this study was performed using invasive methods (OCT and IVUS), this result opens new and promising clinical perspectives for virtual histology evaluation.

Considering the central role of CCTA in defining prognosis and modifying diagnostical-therapeutic strategy in patients with a first diagnosis of CAD, recent evidence proposes anatomical evaluation as the first step in the diagnostic algorithm of patients with suspected CAD [26]. Finally, CCTA is determinant in different settings of CCS: it is able to definitely exclude CAD in patients without atherosclerosis, reassuring patients of the non-ischemic origin of their symptoms and avoiding further instrumental evaluations for at least 5 years [20]; it can reclassify cardiovascular risk among patients with detection of non-obstructive disease, enabling a precision target pharmacological therapy able to modify long term outcome; and it can be the conclusive exam to indicate downstream coronary revascularization (surgical or percutaneous) when significant obstructive (i.e., stenosis > 90%, or stenosis > 50% in “prognostic” vessels) atherosclerosis is detected.

Nevertheless, in the remaining cases, when stenosis of uncertain functional significance [27,28] (i.e., stenosis of 50–90% at visual evaluation) is identified, CCTA is not sufficient per se to directly guide revascularization, and further information with a downstream non-invasive or invasive functional test is required in this setting. This is particularly true when patients with a previous history of revascularization complain of a suspect recurrence of symptoms: in this case, a functional test, such as s-CMR [29], is stronger in predicting revascularization requirement, prognosis, and cost-effectiveness.

### 2.2. Functional Evaluation

As aforementioned, non-invasive functional tests able to allow detection of perfusion abnormalities or wall motion abnormalities under stress were identified as more accurate in CCS than s-ECG, which downgraded it to a lower level of recommendation because of its poor accuracy in detecting CAD, especially in females and in patients with resting ECG abnormalities [30,31].

Instead, anatomical and functional imaging was taken at the same level of recommendation, despite a deeply different kind of information provided: the former on severity and type of atherosclerosis, and the latter on pathophysiological consequences such as ischemia. Recent studies underlined the challenge in detecting the prognostic benefit of an “ischemia-guided” revascularization in CCS.

The ISCHEMIA trial was a multicenter, randomized study that enrolled 5179 patients with optimized medical therapy, documented significant coronary atherosclerosis, and moderate-to-severe inducible ischemia to investigate whether early coronary revascularization (with percutaneous coronary intervention (PCI) or coronary artery bypass graft (CABG)) was superior to medical therapy alone in preventing cardiovascular (CV) events. Medical therapy resulted in a non-inferiority compared to invasive strategy in terms of MACE (CV death, non-fatal MI, unstable angina, heart failure) [4].

Nevertheless, there was a difference in terms of types of events with more “spontaneous” coronary events (Type I and II) in the pharmacological group and more “iatrogenic” (procedure-related) coronary events in the invasively treated group.

A sub-analysis of 2475 subjects with established coronary atherosclerosis showed that CAD severity (defined as atherosclerosis extent and stenosis severity based on the modified Duke prognostic index) was a significant predictor of MACE, irrespective of ischemia severity, which only predicted MI [32].

Although the ISCHEMIA trial failed to show the direct benefit of coronary revascularization in the presence of moderate-severe ischemia, symptoms were reduced in patients revascularized, with less antianginal drug use [4]. There is much evidence to show the importance of revascularization in improving symptoms when ischemia is detected [33], and in patients with established CAD, the functional assessment was demonstrated to improve event-free survival, with respect to sole anatomical evaluation before PCI [34,35,36]. It is still debated whether the prognostic relevance of ischemia is a consequence of the subtending coronary atherosclerosis [37]; in any case, the importance of the imaging functional testing currently available goes beyond the mere detection of ischemia, providing important prognostic information about function, myocardial tissue characterization, and epicardial inflammation, to name a few [38].

#### 2.2.1. Stress Echocardiography

SE is one of the most used second-line stress tests for the assessment of patients presenting with chest pain and intermediate pre-test likelihood of obstructive CAD. It involves performing an echocardiogram at rest and during stress induced by exercise, with the use of inotropic (dobutamine) or vasodilator (dipyridamole or adenosine) drugs [39].

The low cost, absence of ionizing radiation, ease of performance, and good prognostic implications make it one of the most diffuse techniques used to approach patients with CAD. Regional wall motion abnormalities, symptoms, blood pressure response, and ECG changes of the patient are analyzed, both with physical exercise and a pharmacological stressor. However, suboptimal images due to poor acoustic windows and submaximal stress can affect diagnostic accuracy, especially when only regional wall motion abnormalities are investigated [40].

In order to overcome such limitations, in the latest EACVI consensus [41], a new approach with a multiparameter evaluation was proposed, recommending to perform analysis according to a five-step “ABCDE” scheme: step A: analyze regional wall motion abnormalities (RWMA) with 2D echocardiography; step B: B-lines by lung ultrasound (LUS); step C: left ventricle (LV) contractile reserve with volumetric 2D echocardiography; step D: Doppler-based assessment of coronary flow velocity reserve (CFVR) in LAD; and step E: imaging-independent ECG-based assessment of heart rate reserve as heart rate ratio.

This approach was recently validated in a prospective, multicenter study of 3574 patients with CCS, with all-cause mortality being the single endpoint. At a median follow-up of 21 months, this method demonstrated an effective prediction of survival, in particular identifying patients at low risk (ABCDE risk score of 0, the so-called “non-ischemic-dry-strong-warm-fast heart”) and patients at maximum risk (ABCDE risk score of 5, the so-called “ischemic-wet-weak-cold-slow heart”) [42].

#### 2.2.2. Single-Photon Emission Computed Tomography and Positron Emission Tomography

Pharmacologic stress radionuclide myocardial perfusion imaging (MPI) using SPECT or PET are two alternative techniques in the management of patients with suspected or known CAD. 

Stress radionuclide MPI allows visualization of myocardial blood flow or perfusion between the resting and stressed state, allowing identification of ischemic or infarcted areas. This method also provides quantification of left ventricular (LV) volume, ejection fraction (EF), and regional kinetics. SPECT represents one of the most prescribed ischemia tests in CCS patients [43].

After the injection of a myocardial perfusion radiotracer (technetium-99 m), the isotope is extracted from the blood by viable myocytes and retained within the cells for a period dependent on regional blood flow and mitochondrial membrane integrity; therefore, areas with reversible uptake defects (from stress to rest) indicate inducible ischemia, while areas with persistent defects indicate non-viable myocardium [44].

The high sensitivity of this technique is established, and a negative SPECT identifies subjects at low risk of MACE [45].

SPECT challenges include radiation dose, limited quantification of myocardial perfusion, and low spatial and temporal resolution when evaluating an ischemic region [46,47].

Traditional SPECT systems use large sodium iodide crystals, photomultiplier tubes, and parallel-hole collimation, with consequences of a long time for image acquisition, high radiation doses, and suboptimal accuracy [48].

In the last decade, the development of different technological developments, especially the cadmium zinc telluride solid-state detectors, but also the introduction of specialized collimators and software-based resolution recovery [49], increased exam accuracy [50], particularly in patients with poor image quality on traditional cameras, such as in diabetics, obese patients, women, and patients with multiple chronic diseases.

Regardless, significant inter-site variability is still a cogent problem related to this technique, and standardization of acquisition and postprocessing protocols are required to optimize its clinical importance [51]. Absolute quantification of myocardial blood flow (MBF) by PET is emerging as a technique that allows overcoming some limitations, such as the underestimation of myocardial ischemia in challenging settings such as multi-vessel or left-main disease. Recent meta-analyses on the diagnostic performance of non-invasive ischemia tests showed that PET is one of the most effective approaches in detecting functionally relevant CAD [48,52].

In the Danish study of non-invasive testing in coronary artery disease 2 (Dan-NICAD 2) trial, 1732 consecutive symptomatic patients with suspected CAD were enrolled; patients showing coronary stenosis >50% diameter reduction on CCTA were referred for both 3-T CMR and 82Rubidium PET in a randomized order. Functionally significant CAD was identified in approximately 44% of patients by invasive fractional flow reserve (FFR). CMR and PET showed low sensitivities (59% and 64%) but high specificities (84% and 89%) to predict FFR results [53].

Finally, PET is considered the clinical reference standard for the quantification of myocardial perfusion and for evaluating myocardial viability [54]; however, limited expertise and availability of scanners and tracers, high costs, and lower spatial resolution (compared to CT or MRI) [46] represent important limitations to the routine use of the technique.

#### 2.2.3. Stress Cardiac Magnetic Resonance Imaging

Over time, evidence of s-CMR’s central role in the study of CAD has significantly increased.

The increased availability of this technique and the fact that it does not use ionizing radiation and allows precise evaluation of chamber sizes, cardiac function, areas of fibrosis through late gadolinium enhancement (LGE), myocardial perfusion, and myocardial viability, makes it one of the most accurate methods to give prognostic information in CCS patients [55]. The STRATEGY study [29] is a single-center prospective observational trial comparing the cost-effectiveness of functional assessment with s-CMR versus anatomic evaluation with CCTA in 600 consecutive symptomatic patients with a history of previous myocardial revascularization (PCI or CABG) at a mean follow-up of 773.6 ± 345 versus 752.8 ± 291 days, (*p* = 0.21), respectively. Patients with suspected acute coronary syndrome were excluded. The main result was that the evaluation with s-CMR was associated with lower downstream noninvasive and invasive testing (including procedure of revascularization), with a significant reduction of radiation exposure and cumulative costs (59% and 24%, respectively; *p* < 0.001), a lower rate of MACE (5% versus 10%; *p* < 0.010), and a better cost-effectiveness ratio (119.98 ± 250.92 versus 218.12 ± 298.45 Euro/y; *p* < 0.001) compared to CCTA. Different hypotheses were argued to explain these results. 

The thesis of the “iatrogenic” MI as a cardiovascular driver event, as was debated in the results of the ISCHEMIA trial, was reasonably excluded because only 5 of the MIs in the CCTA group and 2 of the events in the CMR group were reported as periprocedural.

Another theory was that the difference in event rates among anatomical and functional strategies could be caused by delayed complications of PCI or CABG, which are not reported as periprocedural events.

Finally, the most probable explanation of the results of the study is that one of the main benefits by which CCTA could improve outcomes is through intensification of medical therapy in patients without a previous history of CAD; while in this setting of patients with previous coronary revascularization, maximal medical therapy should be already set up, explaining why functional evaluation could be more effective [56].

Furthermore, patients with previous revascularization normally have a higher atherosclerotic burden, explaining the sizeable number of abnormal or inconclusive CCTA in this clinical setting, requiring further additional tests. Also, blooming artifacts due to calcified plaques or coronary stents [57] are often responsible for disease overestimation with CCTA [58]. On the contrary, s-CMR provides a binary response (positive or negative), thus not requiring further downstream examinations.

Finally, CCTA was performed in this study with 64-multidetector scanners, currently substituted in many centers by scanners with reduced artifacts and, equally important, a reduced radiation dose [56].

**Figure 2:**
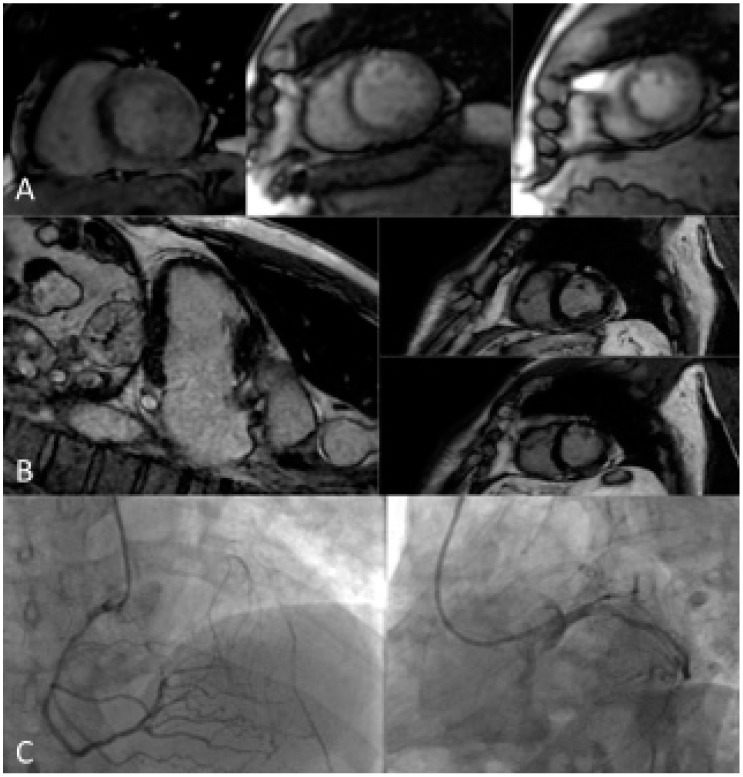
A 63-year-old male patient with a previous history of PCI on LAD, complaining of effort angina, was referred for stress CMR. (**A**) Stress LV SAX perfusion sequences show inducible hypoperfusion in the whole septal segments, anterior medium, inferior medium, and apical segments. (**B**) LGE VLA and SAX sequences show subendocardial ischemic LGE in the anterior medium and apical segments. The area of hypoperfusion extends to a larger territory compared to the LGE area of necrosis. (**C**) ICA shows severe stenosis of proximal RCA (with hetero-coronary collateral circle for LAD territory) and occlusive restenosis of LAD proximal stent. LGE distribution, compared to inducible hypoperfusion, demonstrated viability and ischemia of LAD and RCA territory, worthy of revascularization. CMR: cardiac magnetic resonance; ICA: Invasive coronary angiography; LAD: Left anterior descending; LV: Left ventricle; PCI: Percutaneous coronary intervention; RCA: right coronary artery; VLA: vertical long axis; SAX: short axis.

Figure 2 shows the accuracy of s-CMR in the evaluation of ischemia and viability in a patient with previous percutaneous revascularization and recurrence of angina.

In the IMPACT studies, s-CMR was compared with SPECT, using ICA as the reference standard [59,60].

The MR-IMPACT (Magnetic Resonance Imaging for Myocardial Perfusion Assessment in Coronary Artery Disease) trial was a multicenter, multivendor, double-blind, randomized trial that allowed for the identification of the optimal contrast dose for stress CMR as 0.1 mmol/kg, comparing it with SPECT and using ICA as a reference standard for the diagnosis of CAD. This study demonstrated equal performance of the CMR and SPECT, but with CMR superiority in patients with multi-vessel coronary artery disease [59]. Following these results, the MR-IMPACT II study (Magnetic Resonance Imaging for Myocardial Perfusion Assessment in Coronary Artery Disease Trial II) was conducted. The study was a multicenter, multivendor, double-blind, randomized trial that enrolled 533 patients from 33 centers. All patients underwent ICA, SPECT, and perfusion CMR, with the best protocol for the latter revealed by the previous MR-IMPACT I. 

The study showed that the sensitivity of s-CMR to detect CAD was superior to SPECT (67% vs. 59%), while its specificity was inferior (61% vs. 72%, respectively) [60]. These were the first two large multicenter multivendor trials that have compared s-CMR with other established non-invasive diagnostic techniques, demonstrating it is a safe alternative to SPECT to detect perfusion deficits in CAD. The CE-MARC study [61] also compared s-CMR with SPECT using ICA as a reference standard, demonstrating, in a large population (628 patients) with suspected angina pectoris, that CMR is an alternative to SPECT for the detection of clinically significant CAD, with better sensitivity (86.5% vs 66.5%, *p* = 0.0001) and better negative predictive values (90.5% vs 79.1%, *p* = 0.0001, respectively), but no significant differences in specificity and positive predictive value (*p* = 0.916 and *p* = 0.061, respectively).

The main difference between the CE-MARC and MR-IMPACT trials was the use of a multiparametric CMR protocol in the former; notably, a sub-analysis of the CE-MARC trial showed that the maximum sensitivity for the detection of significant CAD by CMR was provided by the use of all four CMR study components (LV function, myocardial inducible ischemia, myocardial viability by LGE, and coronary magnetic resonance angiography) (86.5%), and, among them, LGE alone had the best accuracy [62]. It is noteworthy that all the aforementioned studies have the limitation of using only qualitative anatomical evaluation of ICA, without functional information as a reference standard.

The study that addressed this gap was the MR-INFORM trial [63], an unblinded, multicenter, clinical-effectiveness trial that enrolled 918 patients with typical angina (and either two or more CV risk factors or a positive exercise treadmill test) by randomly assigning them to either a cardiovascular s-CMR-based strategy or a directly invasive FFR-based strategy.

Revascularization was recommended for patients in the CMR group with ischemia in at least 6% of the myocardium, or in the FFR group with an FFR of 0.8 or less.

Stress perfusion CMR was associated with a lower incidence of coronary revascularization compared to invasively assessed FFR(35.7% of patients vs. 45.0%, respectively) and was found to be non-inferior to ICA-FFR, in terms of the incidence of MACE at a 12-month FU. Notably, only 48.2% of the patients in the CMR group underwent ICA (as compared with 96.8% of those in the FFR group), despite a pretest likelihood for coronary artery disease of 75%. This study represents one of the several demonstrations of the better accuracy of non-invasive imaging for the detection of flow-limiting coronary stenosis compared with invasive functional testing through FFR [27]. Finally, the quantitative myocardial blood flow evaluation through pixel-wise quantitative perfusion mapping has been demonstrated in several small, single-center studies to increase the accuracy of stress perfusion CMR, especially in microvascular dysfunction and multivessel disease [64,65]. The AQUA-MBF (assessment of quantitative MBF) study is a multicenter trial involving 16 centers, with the goal to more systematically evaluate the role of quantitative perfusion evaluation. A recently published sub-study showed the accuracy of this tool in differentiating diseased from healthy myocardium [66]. Despite all the aforementioned positive features of stress CMR, this exam remains one of the most expensive in terms of costs and duration, and it suffers clinical limitations such as claustrophobia and severe artifacts, due to metal devices.

### 2.3. Anatomical Plus Functional Evaluation

Thanks to technological advancements in CCTA over the last decade, two CCTA-based techniques have been developed: s-CTP and FFR_CT_.

Both are non-invasive diagnostic tests that aim to combine precise anatomical information about coronary trees with functional data in a single examination, allowing for a comprehensive evaluation of CAD in both new patients and those who have undergone previous revascularization.

**Figure 3:**
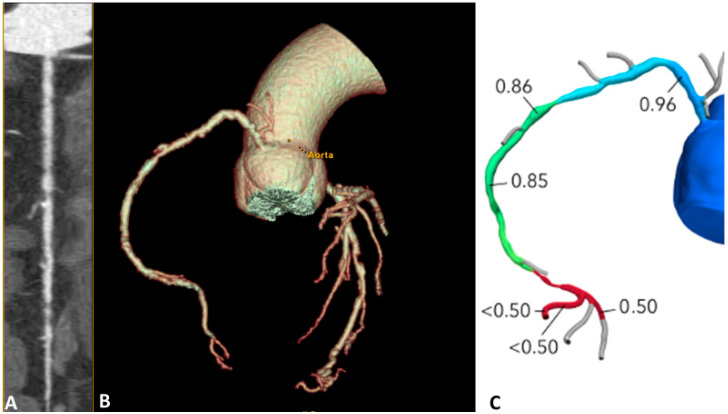
CCTA and FFR_CT_ combined evaluation in a 65-year-old patient complaining of angina. (**A**) The study of the right coronary artery reveals multiple plaques, with stenosis ranging visually from severe to moderate. (**B**) 3D volume rendering reconstruction. (**C**) FFR values are pathological only in the mid-distal segment, indicating hemodynamically significant stenoses at this level. CCTA: Coronary Computed Tomography Angiography; FFRCT: Fractional Flow Reserve Coronary Tomography.

These techniques are based on different pathophysiological assumptions: in s-CTP, a vasodilator stress is used to quantify the MBF; if the myocardium is normally perfused, the contrast agent is regularly transported inside it; if ischemia is present, less contrast agent reaches the affected region and its wash-in or wash-out is delayed. It is possible to measure MBF quantitatively or semi-quantitatively [67,68]. Otherwise, FFR_CT_ uses mathematical models to calculate the FFR providing detailed information on the degree of coronary artery obstruction and the effect such obstructions may have on myocardial perfusion. The main advantage of FFR_CT_ is that it does not require an additional scan time and use of stressors and, therefore, is associated with low radiation exposure. Figure 3 shows the role of FFR_CT_ in defining the functional significance of the stenosis anatomically identified with CCTA. Instead, perfusion imaging is potentially more representative, due to its more physiological nature, but requires additional scan time with higher radiation exposure, as well as the use of a stressor agent [69]. 

S-CTP images can be acquired through static or dynamic protocols: the former provides a purely qualitative assessment and the latter, by the acquisition of multiple datasets, gives a quantitative evaluation by calculating the MBF in every myocardial segment [70], but it is associated with a higher radiation dose. The need to compare these techniques both with pure anatomical CCTA and with other functional imaging techniques arose with the publication of several studies. 

In the ADVANTAGE study, 150 patients with previous stent implantation, referred for non-emergent and clinically indicated ICA due to suspected intrastent restenosis or progression of CAD in native coronary segments, were enrolled. 

In patients with coronary stents, s-CTP significantly improved the diagnostic rate and accuracy of CCTA alone compared with both ICA and invasive FFR as the gold standard, demonstrating once more the supremacy of a functional evaluation in patients already revascularized [71]. The study from Rønnow Sand NP et al., born as a pre-specified sub-study of the Dan-NICAD trial (Danish Study of Non-Invasive Diagnostic Testing in Coronary Artery Disease [72], which was designed to compare the diagnostic performance of SPECT and CMR), and a subgroup of 110 patients with new-onset stable chest pain and one or more coronary stenosis ≥ 50% by CCTA underwent a s-CMR and FFR_CT_ analysis on the CCTA, which were already performed at the time of the original trial. All patients underwent ICA, with the decision on revascularization guided by invasive FFR as a refence of the standard of care. FFR_CT_ exhibited higher sensitivity than perfusion CMR (97% vs. 47%, respectively) in identifying patients requiring coronary revascularization, while stress perfusion CMR demonstrated higher specificity (88% vs. 42% by FFR_CT_) and positive predictive value (67% vs. 47%) [69]. Regarding the comparison between the two functional CCTA-derived methods, Pontone et al. [73] conducted a longitudinal, prospective, consecutive cohort study, enrolling 147 symptomatic patients with suspected CAD, designed to compare the feasibility and accuracy of integrated CCTA+ FFR_CT_ versus that of CCTA + s-CTP for the diagnosis of functionally significant CAD using ICA with invasive FFR as a reference standard. The study confirmed that CCTA alone is sufficient to exclude functionally relevant CAD when obstructive CAD is absent. On the contrary, in the setting of obstructive CAD, both FFR_CT_ or stress CCTA demonstrated equal accuracy in detecting functionally significant stenosis (CCTA + FFR_CT_ sensitivity 88%-CCTA + stress-CCTA sensitivity 92%; *p* = 0.353), and better accuracy than pure anatomical evaluation.

The recently published PRECISE Trial enrolled 2103 patients with stable chest pain, who were randomized to perform testing with a “usual test” (UT) or with a so-called “precision strategy” (PS).

In the UT group site, clinicians chose the initial testing modality, including all the known functional tests or direct catheterization [74].

In the PS group, the PROMISE risk score validated by the previously published PROMISE trial [24] was used to identify patients with minimal risk scores, in which diagnostic testing was deferred, while all the other patients received CCTA with selective FFR-CT when a stenosis of 30% to 90% was anatomically detected.

At one-year follow-up, clinical efficiency was higher with the PS strategy, with lower rates of catheterization without obstructive disease (27 [2.6%]) compared to UT participants (107 [10.2%]; HR, 0.24; 95%CI, 0.16–0.36), while the safety composite of death and MI was similar (HR, 1.52; 95%CI, 0.73–3.15).

**Figure 4:**
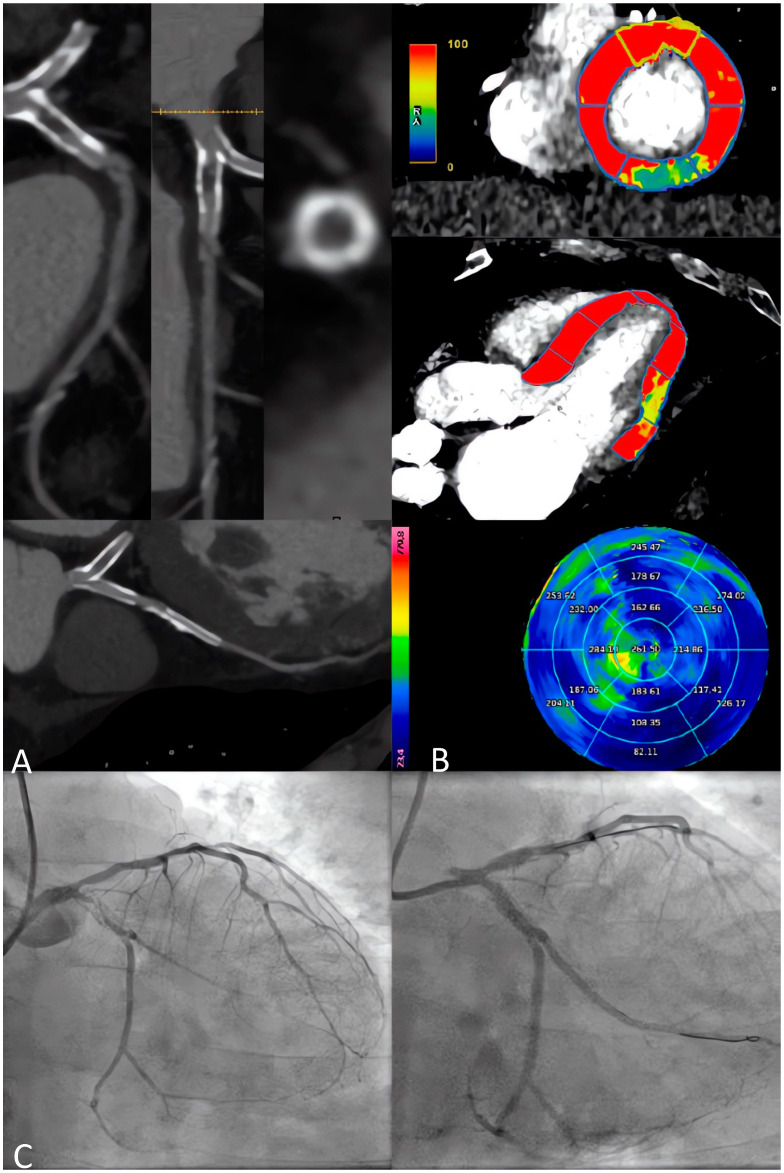
A 65-year-old male patient with a previous history of PCI on LAD and LCx, complaining of the recurrence of effort angina. (**A**) CCTA shows sub-occlusive intrastent re-stenosis of LCx, while the LAD stent appears with some intimal hyperplasia but no severe restenosis. (**B**) The s-CTP with dynamic quantitative perfusion shows an inducible hypoperfusion defect on the inferior-lateral basal segment. (**C**) ICA confirms intrastent sub-occlusive restenosis of LCx treated by PCI, while LAD shows non-severe intrastent restenosis, unworthy of PCI. CCTA: Coronary Computed Tomography Angiography; ICA: Invasive Coronary Angiography; LAD: Left Anterior Descending; LCx: Left Circumflex Artery; PCI: Percutaneous Coronary Intervention.

Despite the promising results in terms of the cost-effectiveness of this strategy, longer follow-up data are required, especially for safety evaluations [74]. Finally, the ongoing CTP-PRO study, an international, multicenter, prospective, open-label, randomized, controlled study is currently evaluating the cost-effectiveness and prognosis of a CCTA+S-CTP strategy versus usual care in intermediate-high-risk patients with suspected or known CAD [75]. Figure 4 shows the accuracy of a combined anatomical and functional evaluation with s-CTP in high-risk patients.

Table 1 describes the main differences among all the abovementioned non-invasive imaging techniques in terms of the type of information provided, radiation exposure, patient medicalization, costs, and current expertise.

## 3. Role of Cardiac Computer Tomography Angiography in the Planning of Myocardial Revascularization

In addition to the aforementioned role of anatomical and functional imaging in the management of CCS, CCTA has sparked significant interest in the realm of preprocedural planning for both percutaneous and coronary revascularization [76].

Cardiac imaging through CCTA is proving valuable, not only for diagnostic purposes, but also in interventional settings for preoperative planning, having received endorsement from the consensus document of the Society of Cardiovascular Computed Tomography [8].

### 3.1. CABG vs. PCI: Role of CCTA in Guiding the Decision-Making

In European and American guidelines, the heart team approach (including cardiac surgeon, interventional cardiologist, and cardiologist) for the decision-making process regarding CABG and PCI is a class I recommendation [2].

CABG surgical revascularization is recognized as the treatment of choice for patients with a more complex CAD, while PCI is preferred when the Syntax Score is lower.

The usefulness of CCTA in helping decision-making has been evaluated in the SYNTAX studies [77,78,79]. In particular, the SYNTAX III revolution study [79], by randomizing two heart teams, showed that treatment decision-making based on CCTA is in almost perfect agreement with the treatment decision derived from ICA in patients with three-vessel CAD. This is an international, multicenter, randomized trial that enrolled 223 patients with invasive or non-invasive angiographic evidence of three-vessel (or equivalent, i.e., patients with hypoplastic RCA in the absence of a right posterior descending artery) and/or left main disease.

Two heart teams (composed of radiologists, interventional cardiologists, and cardiac surgeons) were randomized to receive coronary anatomy information on these patients, based solely on ICA or solely on CCTA, and had to express a first decision on treatment strategy (CABG or PCI or equipoise).

The “CCTA heart team” was asked to express a second decision based on the FFR_CT_ information. Finally, both heart teams were asked to express another decision based on all the available information: ICA, CCTA, and FFR_CT_.

This study introduced novel insights into the application of CCTA as a valuable non-invasive guide for the strategic planning of myocardial revascularization, thanks to its ability to combine, in a single method, stenosis estimation, plaque characterization, and functional assessment when FFR_CT_ was performed.

### 3.2. CCTA in the Planning of PCI

The predictors of PCI success based on anatomy are widely recognized. Optical coherence tomography (OCT) and intravascular ultrasound (IVUS) are imaging methods used to visualize inside blood vessels, offering essential information about lesions and outcomes following stent placement in PCI. However, their invasive nature, high cost, and time-intensive procedures have hindered their widespread use, resulting in varied OCT/IVUS-guided PCI rates among countries and an overall limited penetration rate [80,81].

CCTA is increasingly recognized for its strong correlation with intravascular imaging techniques (both IVUS and OCT) [82,83].

It is recognized as a valuable tool for planning PCI, especially in complex cases such as chronic total occlusions (CTOs) and bifurcations. Furthermore, CCTA has the potential to address procedural hurdles such as incomplete plaque coverage due to inaccurate assessment of lesion length and suboptimal fluoroscopic view angles. Its capability to provide a comprehensive visualization of the coronary vasculature without foreshortening and overlapping further enhances its utility [84]. Additionally, the characterization of plaques through CCTA may contribute to the stratification of the risk of periprocedural complications [85,86,87].

The presence of calcium in plaques, despite being a protective factor regarding vulnerability, has been demonstrated to be associated with stent under-expansion and suboptimal PCI results [87]. Therefore, in vessels identified with a high burden of calcium through CCTA, the use of additional techniques such as rotational or orbital atherectomy and intracoronary lithotripsy with high-pressure balloons may enhance stent expansion [88].

Consequently, awareness of the presence of calcification on CCTA beforehand could potentially impact the outcomes of catheter-based interventions. Furthermore, the presence of hard plaques full of calcium (with CT density > 120 Hounsfield units) was also associated with the more frequent occurrence of dissection or perforation during PCI [89].

On the other side, low-attenuation components have also been linked to periprocedural MI and the no-reflow phenomena. In a post hoc analysis of the SCOT-HEART [90] trial, low-attenuation plaque burden was the strongest predictor of MI, irrespective of CV risk score, coronary artery calcium score, or coronary artery area stenosis. Identifying high-risk plaques on CCTA may assist in adjusting PCI strategy towards direct stenting, the use of distal protection devices, or more potent antithrombotic therapy [91]. As far as concerns CTOs, the percutaneous approach remains a significant challenge for interventional cardiologists. In contrast to ICA, CCTA offers comprehensive visualization of the occlusion site and the distal vessel segment, facilitating the identification of severe calcification, tortuosity, occlusion length, and multiple occlusion sites, all of which contribute to various CT scores (J-CTO or CT RECTOR scores) aimed at predicting the likelihood of success [87,92]. The presence of extensive calcifications in the context of CTO has been widely recognized as a strong independent correlate of unsuccessful PCI, primarily due to guidewire crossing failure. In the study conducted by Sung-Jin Hong et al. [93], the success rate of PCI for CTO increased with the routine use of pre-procedural CCTA. The study revealed that CCTA contributed to a 10% absolute increase in the success rate, along with a reduction in periprocedural coronary perforations and MI. In conclusion, recent literature highlights CCTA as a valuable non-invasive tool for assessing CTO, not only for the selection of patients unlikely to benefit from ICA, but also to provide crucial information for planning the PCI procedure by selecting appropriate devices beforehand, and finally to guide the decision between an antegrade or retrograde approach.

The lesion coverage and appropriately matched vessel diameters are another crucial point in reducing the risk of stent failure and enhancing clinical outcomes in patients undergoing PCI. The rationale for using CCTA in the pre-planning lies precisely in its ability to provide a comprehensive visualization of plaque burden, which is not always achievable with angiography techniques. Pregowski et al. [94] investigated this ability by comparing angiography-guided PCI versus angiography together with CCTA-guided PCI examining immediate procedural outcomes, as defined by IVUS endpoints. Coronary stents selected under CT guidance were significantly longer, with a trend toward a larger nominal stent diameter compared with the angiography-guided strategy alone. This resulted in better lesion coverage and increased stent expansion, supported by a reduction in persistent plaque burden and a tendency towards a larger minimal stent area in the CCTA group.

Finally, the role of FFR_CT_ is to provide evidence in guiding risk stratification and clinical decision-making in candidates for PCI [79]. Still under scrutiny is the ability of CCTA to simulate, in advance, the FFR post-PCI, predicting the hemodynamic impact of stent implantation and providing an additional opportunity to assist interventional cardiologists. To answer this question, the “P3 study” [95] is a prospective, international, and multicenter study of patients with chronic coronary syndromes undergoing PCI whose primary aim is to assess the agreement between the predicted FFR_CT_ post-PCI derived from the planner and invasive FFR.

The enrollment started in February 2019 and has the goal to totally enroll 127 patients, with the ambitious task of providing data to improve patient selection for PCI, anticipating the clinical benefit of the intervention, and refining the revascularization strategy.

The study of Sonck et al., a prospective, international, and multicenter study, tried to validate the accuracy of the FFR_CT_ planner in predicting FFR after PCI by assessing the agreement between the predicted post-PCI FFR_CT_ by the planner and the post-PCI measured FFR. The conclusion is that the accuracy and precision of the FFR_CT_ planner are high and remain high in cases with focal and diffuse disease and with low and high calcium burden [96]. Additionally, the multicenter, randomized P4 trial, currently underway, aims to compare clinical outcomes between two imaging strategies for guiding PCI: coronary CT-guided PCI (investigational technology) and IVUS-guided PCI (comparator). Specifically, for the CT-guided arm, randomized patients will benefit from the use of the FFR_CT_ planner, validated in previous studies, to generate PCI simulations for both pre-operative planning and interactive use during angiography [97]. The current evidence and the future results of the study may lead to new possibilities for utilizing CCTA-based PCI planner software, capable of simulating blood flow and coronary physiology after a virtual PCI. By integrating anatomical information with functional insights, this approach aims to better predict the pre-interventional scenario and guide therapeutic interventional decisions.

### 3.3. CCTA in the Planning of CABG

As previously discussed, the utility of CCTA in the post-operative evaluation of patients undergoing surgical revascularization is acknowledged. The ability of cardiac CT, whether associated with FFR_CT_ or not, to assist in pre-operative planning for patients eligible for surgical revascularization is currently under investigation.

In a pilot survey conducted among surgeons participating in the randomized SYNTAX III Revolution trial, CCTAs and FFRCT results of 20 patients were presented to 5 cardiac surgeons; in 84% of the cases, there was excellent agreement on the number of anastomoses to be made in each patient. Consequently, the feasibility of CABG planning using non-invasive imaging methods is suggested [98]. 

The ongoing FAST-TRACK study aims to explore this aspect. It is a multicenter, prospective study evaluating the feasibility and safety of planning and executing CABG solely based on CCTA combined with FFR_CT_, without knowledge of the anatomy defined by ICA [98].

**Figure 5:**
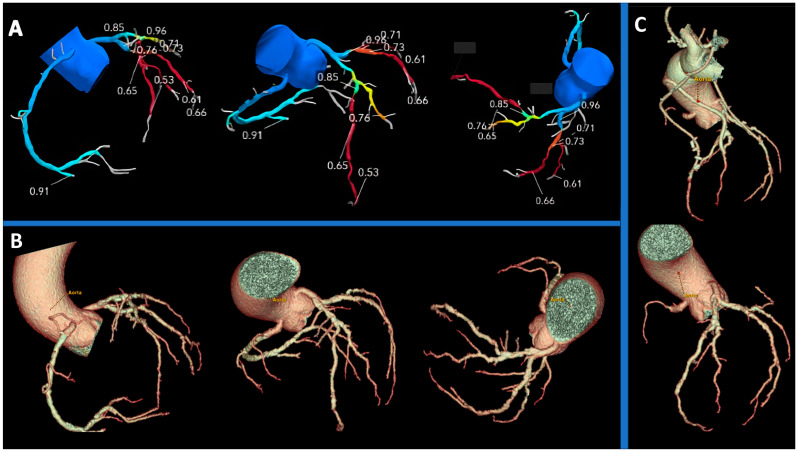
Anatomical evaluation and functional assessment of coronary disease derived from CCTA in a pre-surgical setting. (**A**) FFR-CT 3D model of the coronary tree, with FFR_CT_ values reported: the left anterior descending and circumflex coronary artery exhibit pathological FFR values (<0.8), while the first diagonal branch demonstrates values close to pathological thresholds. (**B**) 3D volume rendering reconstruction of the coronary tree provides an assessment of the anatomy in a pre-surgical scenario. (**C**) 3D volume rendering reconstructions are used to compare graft points decided preoperatively based on FFR values, with post-CABG (upper image) and pre-CABG (lower image) representations. CCTA: Coronary Computed Tomography Angiography; FFRCT: Fractional Flow Reserve Coronary Tomography; CABG: Coronary Artery Bypass Graft.

The FAST-TRACK study’s outcome could redefine the role of CCTA and FFRCT in pre-operative contexts, providing crucial insights into their efficacy for guiding myocardial revascularization planning, as shown in the example in Figure 5.

## 4. Limitations

Despite now being integrated into guidelines, the approach to patients with CCS using non-invasive imaging, in particular for CT and MR-based methods and second-level stress imaging techniques, currently has usage limitations.

In more rural and peripheral contexts, there are currently neither dedicated personnel for CT and MRI-based cardiac imaging nor high-performance technologies that allow adherence to the most recent guideline recommendations [99].

Table 1 shows a comparison between non-invasive imaging techniques in CCS evaluation, highlighting how, for CT and MR-based methods, the technological commitment ranges from medium to high, and the competency required to perform the examination is high.

This type of approach, in fact, requires not only technological effort but also significant expertise.

An expert in cardiac imaging should be capable not only of the optimal application of imaging tests but also of the appropriate execution of the exam, considering the dose of exposure and the prescription and administration of cardioactive drugs (nitrates, beta-blockers, and vasodilator stress agents), including indications and contraindications of the abovementioned drugs in different clinical settings.

Moreover, descriptive capabilities alone are not sufficient: clinical skills are also necessary for the appropriate interpretation of imaging based on the clinical context.

Even in medium-sized centers where technologies are state-of-the-art, the use of stress CT and MR methods is not widespread due to organizational difficulties, budget constraints, and reconciliation issues, including legislative ones, among various specialties (cardiologists, radiologists, nuclear medicine physicians).

In Europe, there are currently tertiary cardiac imaging centers, either in conjunction with other specialties (such as radiology) or as stand-alone departments, which are driving this type of imaging-based approach. These centers are also providing models for future organizational development and approaches based on competencies rather than medical specialization [100].

The implementation of specific training programs to expand the pool of experts in cardiac imaging and the increasingly widespread adoption of teleradiology offer hope for a progressive reduction of these limitations, providing all patients with standardized diagnostic approaches in line with the guidelines.

## 5. Conclusions

CCS is one of the most common cardiovascular causes of morbidity and uses of medical resources, and the correct management, both in the diagnostic pathway and in the therapeutic strategy, remains still an argument of debate, also considering many different types of scenarios included in this syndrome. 

The role of non-invasive imaging continues to grow in importance, and the progressive use of new techniques and technologies provides a wide range of choices in different settings but also raises doubts about the right strategy to use case-by-case.

In parallel, the role of CCTA in periprocedural planning is promising to acquire more space, both in defining the correct strategy of interventional procedure and in guiding it, by improving patient selection and predicting the possible benefit of the intervention. 

Finally, the establishment of specialized training programs to increase the number of experts in cardiac imaging, combined with the growing adoption of teleradiology, is raising expectations for the broader application of a non-invasive imaging-based approach.

## Figures and Tables

**Table 1:** Comparison between non-invasive imaging techniques in CCS evaluation in terms of the type of information provided (anatomical, functional, and/or further prognostic information); radiation exposure; patient medicalization required for the imaging performance (beta-blockers, nitrates, stressor agents); costs and current expertise. In Patient Medicalization, “+/−” indicates that, in some cases, it is possible to perform the investigation without the use of drugs. CCS: Chronic Coronary Syndromes; CCTA: Cardiac Computer Tomography Angiography; FFRCT: Fractional Flow Reserve derived from CCTA datasets; PET: Positron Emission Tomography; s-CMR: Stress Cardiac Magnetic Resonance Imaging; s-CTP: Stress Computed Tomography Perfusion; SE: Stress Echocardiography; SPECT: Single-photon Emission Computed Tomography.

	SE	SPECT	PET	s-CMR	CCTA	CCTA + FFR_CT_
Anatomical information	−	−	−	−	+	+
Functional Information	+	+	+	+	−	+
Further prognostic information	LV volumes and function, functional capacity	LV volumes and function	LV volumes and function, pericoronary inflammation	LV and RV volumes and function, myocardial tissue characterization (edema, LGE)	Pericoronary inflammation. LV and RV volumes and function, myocardial tissue characterization (specific protocols required)	
Radiation Exposure (mSv)	−	++	++	−	+	+
Patient Medicalization	+/−	+	+	+	+/−	+/−
Costs	Low	Medium	Very High	Medium-High	Medium	Very High
Current expertise	High	High	Low	Medium	Medium	Low
